# X Chromosomes Alternate between Two States prior to Random X-Inactivation

**DOI:** 10.1371/journal.pbio.0040159

**Published:** 2006-05-09

**Authors:** Susanna Mlynarczyk-Evans, Morgan Royce-Tolland, Mary Kate Alexander, Angela A Andersen, Sundeep Kalantry, Joost Gribnau, Barbara Panning

**Affiliations:** **1**Department of Biochemistry and Biophysics, University of California San Francisco, San Francisco, California, United States of America; **2**Department of Genetics and the Carolina Center for the Genome Sciences, University of North Carolina Chapel Hill, Chapel Hill, North Carolina, United States of America; **3**Department of Cell Biology, Erasmus University Medical Center, Rotterdam, Netherlands; Stowers Institute for Medical ResearchUnited States of America

## Abstract

Early in the development of female mammals, one of the two X chromosomes is silenced in half of cells and the other X chromosome is silenced in the remaining half. The basis of this apparent randomness is not understood. We show that before X-inactivation, the two X chromosomes appear to exist in distinct states that correspond to their fates as the active and inactive X chromosomes. *Xist* and *Tsix,* noncoding RNAs that control X chromosome fates upon X-inactivation, also determine the states of the X chromosomes prior to X-inactivation. In wild-type ES cells, X chromosomes switch between states; among the progeny of a single cell, a given X chromosome exhibits each state with equal frequency. We propose a model in which the concerted switching of homologous X chromosomes between mutually exclusive future active and future inactive states provides the basis for the apparently random silencing of one X chromosome in female cells.

## Abstract 2

During female mammalian development, one of the two X chromosomes in each cell is inactivated. Prior to X-inactivation, these chromosomes appear to exist in distinct states that correspond to their fates.

## Introduction

At least 10% of mammalian genes are transcribed from only one allele that, in most instances, is chosen at random [ 1]. The mechanisms for achieving differential regulation of homologous alleles in a stochastic manner are poorly understood [ 2]. X-chromosome inactivation in mammals is an example of random monoallelic expression; the majority of genes on one X chromosome in XX females are silenced to equalize expression of X-linked genes with XY males [ 3]. Understanding the process by which the two X chromosomes are assigned active and inactive fates in a stochastic manner will provide insight into how randomness can be achieved in other biological contexts.

X-inactivation is a well-defined system for studying the mechanisms that generate the randomness of monoallelic expression. In the earliest stages of female embryogenesis, X-linked genes exhibit biallelic expression in female cells. Upon receipt of a developmental cue, one X chromosome is silenced in half of the cells of the embryo and the other X chromosome is silenced in the remaining half. Once the identities of the active and inactive X chromosomes (Xa and Xi) are established, they are stably propagated throughout all subsequent cell divisions [ 4]. Female mouse embryonic stem (ES) cells provide a model system to study the mechanisms that control the initial, stochastic determination of the Xa and Xi in vitro. As in the pluripotent cells of the early embryo, genes are expressed from both X chromosomes in female ES cells. X-inactivation can be induced in vitro, recapitulating the random silencing process that occurs in vivo during differentiation [ 5]. In addition, genetic elements have been identified that affect the randomness of X-inactivation [ 6, 7], making female ES cells a useful tool for the study of the mechanisms that control random monoallelic expression.

The *X-inactivation center (Xic)* is a master *cis*-regulatory element on the X chromosome that controls X-inactivation [ 4]. The *Xic* contains a number of elements, including *Xist* and *Tsix,* a sense/antisense pair of noncoding RNAs that are transcribed from both X chromosomes prior to X-inactivation [ 8– 10]. When embryonic cells differentiate and X-inactivation is initiated, *Xist* RNA spreads in *cis* from the *Xic* to coat and silence one X chromosome [ 9, 10]. *Xist* and *Tsix* play a role in the stochastic determination of which X chromosome will become the Xi and which will become the Xa. In cells heterozygous for either an *Xist* or a *Tsix* mutation *,* X-inactivation is nonrandom: an *Xist* mutant chromosome always becomes the Xa [ 11, 12] and a *Tsix* mutant chromosome always becomes the Xi [ 13– 15]. Heterozygous mutations of *Xist* or *Tsix* also exhibit effects in *trans:* not only are the fates of the mutant X chromosomes fixed but also the fates of the wild-type X chromosomes are determined. Thus, information about the fate of one X chromosome must be transmitted to the other X chromosome, ensuring that decisions about their fates are coordinated and that there is random and exclusive silencing of one X chromosome in female cells. The manner in which the opposing activities of *Xist* and *Tsix* regulate the fates of both X chromosomes in female cells remains mysterious.

In this paper, we present evidence that in individual pluripotent embryonic cells that are poised for X-inactivation, the X chromosomes exist in two mutually exclusive states. In heterozygous *Xist* and *Tsix* mutant cells, these states predict the fates of the X chromosomes, indicating that one X chromosome adopts a future Xa state and the other X chromosome adopts a future Xi state. In wild-type cells, X chromosomes switch between these states such that, among the progeny of a single cell, a given X chromosome exhibits each state with equal frequency. Thus, the concerted switching of homologous X chromosomes between mutually exclusive future Xa and future Xi states may provide the basis for the apparent randomness of X-inactivation.

## Results

### X-Chromosomal Loci Show a High Frequency of Singlet/Doublet Fluorescence In Situ Hybridization Signals in ES Cells

**Figure 1:**
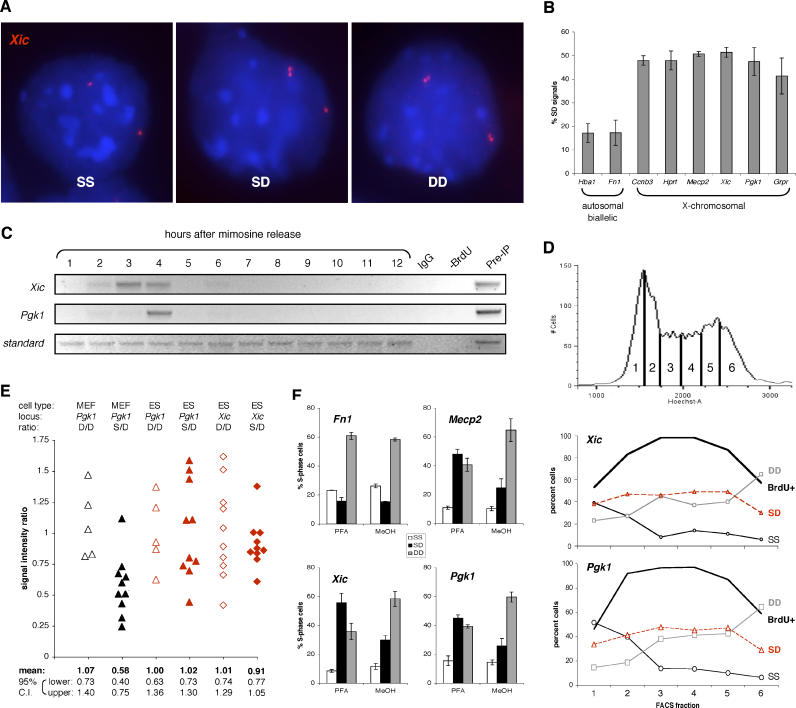
X-Chromosomal Loci Display SD FISH Signals Independent of Asynchronous DNA Replication (A) FISH for *Xic* genomic sequences (red) demonstrates the three classes of signals in PFA-fixed female ES cells. DNA was stained with DAPI (blue). (B) ES cells display an elevated proportion of SD signals at X-chromosomal loci. Average data from two to four experiments ( *n* > 150), scored by two independent scorers, are presented. Error bars indicate one standard deviation. See Figure S1A for complete scoring of SS, SD, and DD signals in these cells. (C) The *Xic* and *Pgk1* show a single peak of replication in female ES cells. DNA was isolated from cells that were arrested in G1, released into S phase, and pulsed with BrdU at hourly intervals. Progression through S phase was monitored by FACS ( Figure S1B). An equal amount of BrdU-labeled human DNA was added to each time point. BrdU-labeled DNA was immunopurified, and sequences present in each fraction were assessed by PCR. Standard shows amplification of a human sequence, as a control for variability in immunoprecipitations. IgG represents PCR analysis from labeled DNA purified with mouse IgG instead of anti-BrdU antiserum. –BrdU indicates analysis of an anti-BrdU immunoprecipitation from unlabeled DNA. Pre-IP depicts PCR analysis of the input DNA. (D) Live female ES cells, pulse-labeled with BrdU, were sorted into six fractions by Hoechst staining for DNA content (upper panel). FISH for the *Xic* (middle panel), and *Pgk1* (lower panel) in these fractions shows constant, high proportions of SD signals (red triangles) throughout S-phase. Proportions of SS (black circles) and DD (gray squares) are also shown. The high proportion of BrdU-positive cells in all fractions (bold black line) shows that a substantial proportion of cells in all six fractions are in S phase. Over 80% of cycling ES cells are in S phase and fewer than 10% are in G1 [ 40], inevitably leading to the inclusion of early S phase cells in fraction 1. Data are representative of two or three independent experiments. (E) In ES cells, singlet and doublet FISH signals for X-chromosomal loci exhibit equivalent fluorescence intensity. Plots show the ratios of S/D (solid symbols) or D/D (open symbols) FISH signal intensities in individual MEF or ES cell nuclei displaying an SD or DD pattern for *Pgk1* or the *Xic* as indicated. The intensity of both pinpoints in each doublet was summed to calculate the total intensity of doublet signals. When calculating the D/D intensity ratios, the two doublets in a cell were randomly assigned to the numerator or denominator. Mean ratio values and 95% confidence intervals for the means are indicated. (F) Comparison of the proportions of cells displaying SS (white), SD (black), and DD (gray) signals for an autosomal locus *(Fn1)* and three X-chromosomal loci *(Mecp2, Xic, and Pgk1)* in S-phase ES cells upon PFA or MeOH fixation.

While using fluorescence in situ hybridization (FISH) to visualize the *Xic* in female ES cells fixed with paraformaldehyde (PFA), we observed that a high frequency of cells displayed a single pinpoint FISH signal at one allele and a double pinpoint at the other. To determine whether this feature was unique to the *Xic,* we analyzed a number of other X-chromosomal loci. Cells were scored as showing a singlet signal for each allele (SS), a doublet signal for each allele (DD), or a pattern in which one allele appeared as a singlet and the other as a doublet (SD) ( Figure 1A). For all X-chromosomal loci examined, the SD signal class was the most abundant, comprising 40% to 50% of the population ( Figures 1B and S1A). This proportion is significantly greater than the fraction of SD signals observed for two autosomal loci, which exhibited the SD pattern in fewer than 20% of cells ( Figures 1B and S1A). Thus, the high proportion of cells displaying SD signals is a unique feature of X-linked sequences.

A high frequency of SD FISH signals can be indicative of asynchronous replication of the two alleles, as singlet and doublet signals can reflect unreplicated and replicated loci respectively [ 16]. However, a singlet FISH signal can also occur at a replicated locus [ 17]. To determine whether the high proportion of cells exhibiting SD signals was due to asynchronous replication, we directly measured the replication timing of X-linked sequences. ES cells were blocked in G1 and released into S phase. At hourly intervals, cells were pulsed with 5-bromo-2-deoxyuridine (BrdU) to label replicating DNA. BrdU-containing DNA was immunopurified from each time point and assayed by PCR for X-linked sequences. The *Xic* and *Pgk1* each showed a single peak of BrdU incorporation early in S phase ( Figure 1C). Therefore, neither X-linked locus was subject to highly asynchronous DNA replication. We next performed FISH for the *Xic* and *Pgk1* in female ES cells sorted by DNA content. The proportion of cells exhibiting SD signals increased at the beginning of S phase, remained fairly constant throughout S phase, and decreased at the end of S phase ( Figure 1D) *.* Taken together, these data demonstrate that even though both alleles of each of these X-chromosomal loci replicated early, SD signals persisted throughout S phase. Furthermore, they suggest that the singlets in cells exhibiting SD FISH signals are replicated alleles.

If a singlet signal in a cell showing an SD signal pattern represents a replicated allele, then it should contain the same amount of DNA as the two pinpoints comprising the doublet signal. To test this hypothesis, we determined the relative fluorescence intensity of singlet and doublet signals for the *Xic* and *Pgk1*. We validated this assay on X-linked sequences in female fibroblasts, in which singlet FISH signals correspond to unreplicated loci and doublet FISH signals correspond to replicated alleles [ 18]. In female mouse embryo fibroblasts (MEFs) exhibiting DD signals for *Pgk1,* the distribution of D/D intensity ratios centered around 1 ( Figure 1E), demonstrating that two replicated alleles display equal signal intensities. We next compared the intensity of the singlet to the sum of the two pinpoints in the doublet (S/D) in female MEFs exhibiting SD signals for *Pgk1*. The S/D intensity ratios centered around 0.5 ( Figure 1E), indicating that relative fluorescence intensity can be used to measure a two-fold difference in DNA content. Quantification of FISH signals can therefore be used to assay differences in DNA content at individual loci in single cells. In ES cells, the ranges of S/D intensity ratios for both the *Xic* and *Pgk1* were very similar to the ranges of D/D ratios: in both cases, the distributions centered approximately around 1 ( Figure 1E). These results indicated that the singlet and doublet FISH signals in ES cells exhibiting an SD pattern for the *Xic* and *Pgk1* contained the same amount of DNA. In combination, these analyses demonstrate that the unusually high proportion of ES cells displaying SD signals for X-chromosomal loci reflects something other than asynchronous DNA replication. We refer to the high frequency of PFA-fixed cells exhibiting SD signals that are Independent of Asynchronous DNA Replication as SIAR.

In a recent study, it was suggested that nuclear organization was important for replicated sequences to appear as singlet FISH signals [ 17]. To determine whether SIAR required an intact nucleus, we compared the proportion of ES cells exhibiting SD signals upon PFA fixation to that seen upon methanol:acetic acid (MeOH) fixation. While PFA fixation preserves the three-dimensional organization of the nucleus by cross-linking nucleic acids and proteins, MeOH fixation destroys nuclear architecture by extracting the bulk of histones and other chromatin proteins [ 19]. The distribution of SS, SD, and DD signals for an autosomal gene, *Fn1,* did not differ significantly between fixation methods ( Figure 1F). In contrast, these two fixation conditions resulted in different distributions of FISH signals for the X-chromosomal loci *Mecp2, Pgk1,* and the *Xic*. For these loci, the proportion of cells displaying SD signals decreased while the proportion of cells exhibiting DD signals increased in MeOH-fixed samples compared to PFA-fixed samples ( Figure 1F). The changes in the relative proportions of cells exhibiting SD and DD signals indicate that when nuclear structure is disrupted, some replicated X-chromosomal loci that would appear as singlets in an intact nucleus resolve into doublets. In addition, these results suggest that native chromatin structure and/or nuclear organization is necessary for some replicated alleles of X-chromosomal loci to appear as singlets.

### X Chromosomes Differ prior to X-Inactivation

**Figure 2:**
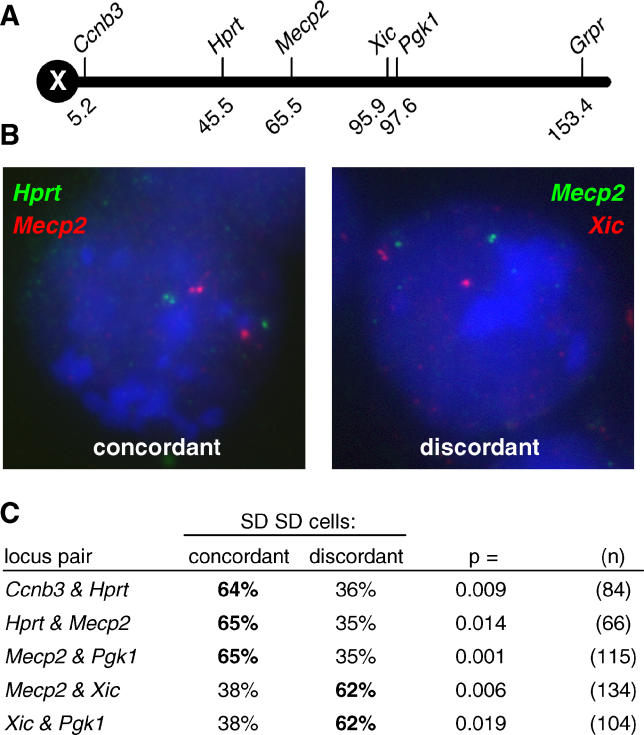
X Chromosomes Differ from One Another in ES Cells (A) Map of the X chromosome showing positions (Mb) of loci assayed for SIAR. (B) Concordant *Mecp2* (red) and *Hprt* (green) (left) and discordant *Xic* (red) and *Mecp2* (green) (right) FISH signals. (C) Frequencies of concordance and discordance for specified locus pairs in ES cells. *p*-Values, determined using a χ ^2^ test, reflect the probability that the observed distributions are random.

All X-chromosomal loci examined ( Figure 2A) exhibited SIAR in female ES cells. We tested whether the singlet FISH signals for multiple loci appeared on the same chromosome or were randomly distributed between the two X chromosomes. Closely linked loci were analyzed pairwise and, among the cells that exhibited SD signals for both probes, the proportion in which singlet signals occurred on the same chromosome (concordant signals) was determined ( Figure 2B). *Ccnb3* and *Hprt, Hprt* and *Mecp2,* and *Mecp2* and *Pgk1* each exhibited approximately 65% concordant signals ( Figure 2C). This 65% concordance is significantly higher than the 50% concordance that would be expected for a random distribution of singlet signals between chromosomes ( *p* < 0.02), indicating that the behavior of loci on each X chromosome is coordinated. One X chromosome exhibits a higher frequency of singlet signals along its length, and the other X chromosome exhibits a higher frequency of doublet signals. These results suggest that, even though X-inactivation has not yet occurred and X-linked genes are biallelically expressed, the two X chromosomes in female ES cells already differ from each other.

When carrying out pairwise analysis of X-chromosomal loci, we found that the *Xic* was unusual in that it was oppositely coordinated with adjacent genes. *Mecp2* and the *Xic,* and the *Xic* and *Pgk1* exhibited a bias against concordant signals, displaying concordance in only 38% of cells scored ( Figure 2C). The *Xic* contains *Xist,* which is unusual in that it is the only gene expressed exclusively from the Xi. The opposite behavior of the *Xic* relative to other X-chromosomal loci in ES cells therefore parallels the opposite expression patterns of *Xist* and X-linked genes after X-inactivation. This parallel suggests a relationship between the appearance of the X chromosomes by FISH prior to X-inactivation and their fates as the Xa and Xi.

### *Xist* and *Tsix* Control SIAR and X Chromosome Fate

To determine if there was a correlation between the appearance of the X chromosomes by FISH prior to X-inactivation and their fates after X-inactivation, we analyzed SIAR in ES cell lines that will undergo nonrandom X-inactivation. If such a correlation exists, then the identities of the X chromosomes displaying singlet and doublet FISH signals should be nonrandom in ES cell lines that are poised for nonrandom X-inactivation. In ES cells heterozygous for an *Xist* mutant chromosome (Δ *Xist*/+), the wild-type X chromosome is always silenced and the mutant chromosome always remains active upon X-inactivation [ 11, 20]. In ES cells bearing a *Tsix* mutant chromosome ( *Tsix*-pA/+), the wild-type X chromosome remains active and the mutant chromosome is inactivated upon X-inactivation [ 14].

**Figure 3:**
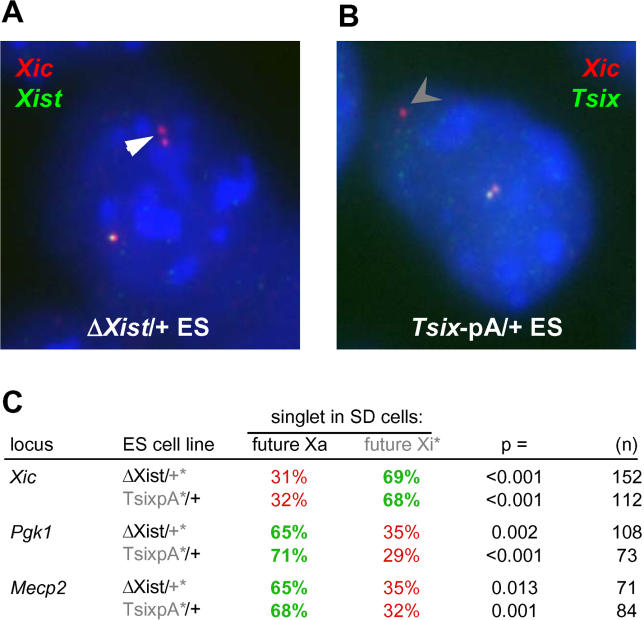
The Future Xa and Future Xi Exhibit Distinct Frequencies of Singlet FISH Signals (A) Allele-specific FISH for the *Xic* (red) in Δ *Xist*/+ ES cells. An *Xist* probe (green) identifies the wild-type allele. White arrowhead indicates the Δ *Xist* allele. (B) Allele-specific FISH for the *Xic* (red) in *Tsix*–pA/+ ES cells. *Tsix* RNA (green) identifies the wild-type allele. Grey arrowhead indicates the *Tsix*–pA allele. (C) Table summarizing scoring of allele-specific FISH in Δ *Xist*/+ and *Tsix*–pA/+ ES cells. For three X-chromosomal loci, SD cells were scored for identity of the allele displaying the singlet signal. The X chromosome indicated in black always becomes the Xa, and that in gray and marked with an asterisk always becomes the Xi. The allele indicated in green will be the expressed allele after X-inactivation and the allele indicated in red will be the silent allele. *p*-Values reflect the probability that the observed distributions are random.

We performed allele-specific FISH for X-chromosomal loci in Δ *Xist*/+ ES cells ( Figure 3A) or *Tsix*-pA/+ ES cells ( Figure 3B). The *Xic, Pgk1,* and *Mecp2* were scored individually in both cell lines. While the overall proportions of SS, SD, and DD signal patterns for these loci did not differ between wild-type ES cells and *Xist* and *Tsix* mutant ES cells (unpublished data), the identities of the alleles displaying the singlet and doublet FISH signals in cells with SD signals were nonrandom in the mutant cell lines. In both Δ *Xist*/+ and *Tsix*-pA/+ cell lines, the X chromosome that will become the Xi exhibited singlet signals for the *Xic* at a high frequency (approximately 70% of SD cells; Figure 3C) and singlet signals for *Pgk1* or *Mecp2* at a low frequency (approximately 30% of SD cells; Figure 3C). The future Xa showed the opposite patterns ( Figure 3C). These results demonstrate that *Xist* and *Tsix* mutations affect SIAR and that, prior to nonrandom X-inactivation, the future Xi and future Xa show different probabilities of exhibiting a singlet signal for the *Xic* and other X-chromosomal loci.

X-chromosomal loci showed distinct frequencies of FISH signal patterns on the future Xa and future Xi in *Xist* and *Tsix* mutant ES cells, supporting the idea that the two X chromosomes adopt distinct states prior to nonrandom X-inactivation. One X chromosome exists in a future Xi state, which causes singlet FISH signals to occur at a high frequency at the *Xic* and at a low frequency at other X-linked sequences. The second X chromosome adopts the future Xa state, which causes singlet FISH signals to occur at a low frequency at the *Xic* and at a high frequency at other X-chromosomal loci.

### X Chromosomes Alternate between Two States prior to Random X-Inactivation

The coordination of SIAR in wild-type ES cells ( Figure 2C) indicated that the two X chromosomes existed in two distinct states similar to those observed in *Xist* and *Tsix* mutants. We hypothesized that in cells poised for random X-inactivation, these X chromosome states might also be indicative of X chromosome fates. Two predictions arise from this hypothesis. First, each X chromosome in wild-type ES cells should exist in the future Xi state in the same proportion of cells in which that chromosome will be inactivated. Second, because the fate of each X chromosome is not yet fixed in wild-type ES cells, the state of each X chromosome must not be fixed either.

**Figure 4:**
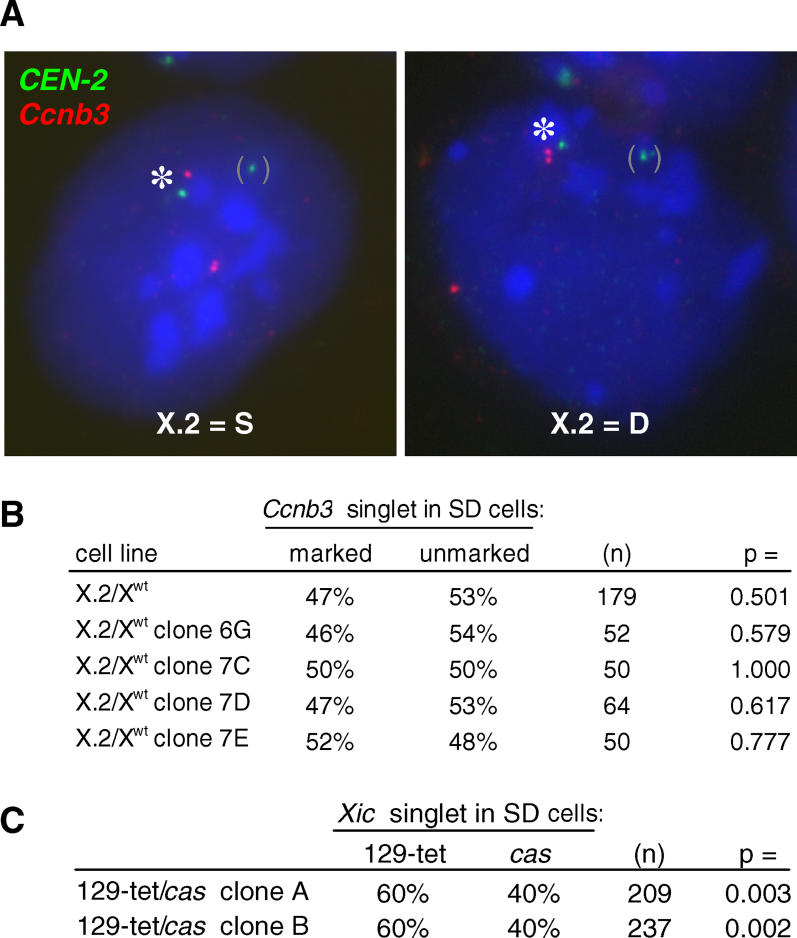
X Chromosomes Appear to Switch between States (A) The two SD signal configurations observed for *Ccnb3* (red) by allele-specific FISH in X.2/X ^wt^ ES cells, in which one X chromosome is fused to Chromosome 2. The marked allele (asterisk) is scored by its proximity to a *CEN-2* probe (green). The *CEN-2* signal on wild-type Chromosome 2 is indicated by parentheses. (B) Allele-specific scoring of *Ccnb3* in X.2/X ^wt^ ES and four single cell-derived clones. Nonsignificant *p*-values indicate a random distribution. (C) Scoring of allele-specific FISH for the *Xic* ( Figure S2) in two independently derived 129-tet/ *cas* ES cell lines **.** *p*-Values indicate that the *Xic* on the 129 chromosome exhibits a singlet signal at a higher frequency than would be expected by random chance.

In an ES cell line that will undergo random X-inactivation, one X chromosome should exist in the future Xi state in half of the cells in the population and the other X chromosome should exist in the future Xi state in the remaining half. By FISH, an X-linked gene should appear as a singlet on one X chromosome in 50% of SD cells and on the other X chromosome in the remaining 50%. To test this prediction, we performed allele-specific FISH in X.2/X ^wt^ ES cells, which are poised to undergo random X-inactivation [ 21]. In these cells, one X chromosome is marked by a centromeric fusion to Chromosome 2 [ 21]. *Ccnb3,* an X-chromosomal locus that exhibited SIAR ( Figure 1B) and is closely linked to the fusion point, was analyzed in combination with a probe proximal to the centromere of Chromosome 2, which identified the X.2 chromosome ( Figure 4A). The X.2 chromosome and the wild-type X chromosome each exhibited a singlet signal for *Ccnb3* in approximately 50% of SD cells ( Figure 4B), consistent with the marked X chromosome existing in the future Xi state in half of the cells and the wild-type X chromosome adopting this state in the remaining half.

X-inactivation is partially skewed in cells that are heterozygous at the *Xce,* an X-linked control element that influences randomness of X-inactivation [ 22]. We analyzed ES cell lines containing X chromosomes that carry different *Xce* alleles to determine whether the frequency with which a chromosome adopts each state correlates with the degree of skewing observed upon X-inactivation. In ES cells heterozygous for M. musculus 129 and M. castaneus Ei X chromosomes (129/ *cas*), the 129 X chromosome will be inactivated in approximately 80% of cells, and the *cas* X chromosome will be inactivated in the remaining 20% [ 7, 23]. Allele-specific FISH for the *Xic* ( Figure S2) was performed in two independent 129-tet/ *cas* ES cell lines in which the *Xic* allele on the 129 X chromosome is marked by a tet-operator array integration that does not disrupt the *Xce* effect [ 11]. Based on the frequency with which the future Xi exhibited a singlet FISH signal in mutant cells destined for nonrandom X-inactivation, we calculated that in 129/ *cas* ES cells, the *Xic* on the 129 allele should appear as a singlet in approximately 62% of SD cells ( Figure S3). Consistent with this prediction, the singlet appeared on the 129 allele in 60% of SD cells ( Figure 4C). Together, analysis of ES cell lines that undergo completely random, completely nonrandom, and skewed X-inactivation suggests that there is a relationship between the state of an X chromosome in ES cells and its fate as the Xa or Xi.

The fates of the X chromosomes are not fixed in wild-type ES cells, as a population of ES cells derived from a single progenitor cell undergoes random X-inactivation upon differentiation [ 11, 24]. If the states of the X chromosomes in ES cells reflect their fates upon X-inactivation, the states should not be fixed in ES cells that undergo random or skewed X-inactivation. To test this hypothesis, we first analyzed four clonal derivatives of the X.2/X ^wt^ ES cell line, each of which is subject to random X-inactivation (unpublished data). In each clone, the X.2 chromosome exhibited a singlet signal for *Ccnb3* in approximately half of cells displaying SD signals ( Figure 4B). Thus, each clone recapitulated the pattern observed in the parental cell line. In the two clonally derived 129-tet/ *cas* ES cell lines, the *Xic* on the 129 chromosome also appeared as a singlet in some cells exhibiting SD signals and a doublet in others ( Figure 4C). Thus, the marked chromosome in each cell line, which would have existed in either the future Xa or the future Xi state in the founding cell of each clone, assumed the future Xa state in some daughter cells and the future Xi state in others. Therefore, the states of the X chromosomes cannot be fixed; rather, the two X chromosomes must switch between states in cycling ES cells.

### SIAR Is Restricted to Cells Poised for Random X-Inactivation

**Figure 5:**
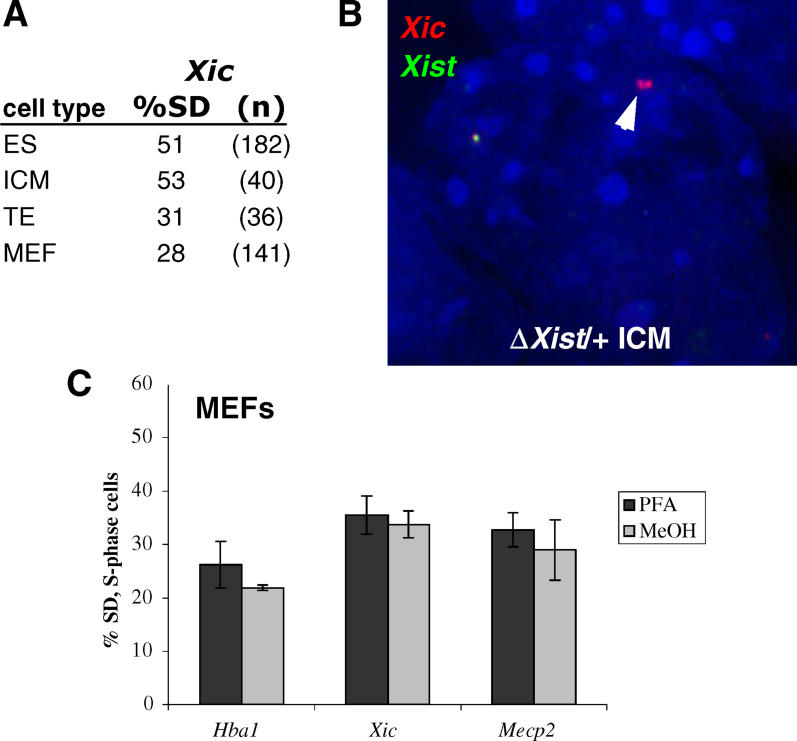
SIAR Is Specific to Pluripotent Cells In Vitro and In Vivo (A) Percent SD signals observed by FISH for the *Xic* in cell types that are poised for X-inactivation (ES and ICM), and in trophectoderm (TE) cells and MEFs, which have completed X-inactivation. (B) Allele-specific FISH for the *Xic* (red) in Δ *Xist*/+ ICM cells. An *Xist* probe (green) identifies the wild-type allele; white arrowhead indicates the Δ *Xist* allele. In 12 of 15 SD cells ( *p* = 0.02), the Δ *Xist* allele exhibited the doublet signal. (C) Percent SD signals observed for an autosomal biallelic locus *(Hba1)* and two X chromosomal loci *(Mecp2* and the *Xic)* in MEFs fixed with PFA (dark gray) or MeOH (light gray). Average data from two experiments ( *n* ≥ 150) are presented; error bars indicate one standard deviation. See Figure S4B for complete scoring of SS, SD, and DD signals.

If future Xi and future Xa states underlie random X-inactivation, then these states should be observed in vivo. We performed FISH for the *Xic* in blastocyst-stage female mouse embryos. Cells from the inner cell mass (ICM) of these embryos are poised for random X-inactivation [ 25]. The percentage of ICM cells exhibiting SD signals for the *Xic* was comparable to that seen in ES cells ( Figure 5A), suggesting that ICM cells also display SIAR. Allele-specific FISH for the *Xic* in Δ *Xist*/+ blastocysts ( Figure 5B) indicated that the wild-type X chromosome, which will become the Xi, more frequently exhibited a singlet signal, and the mutant X chromosome, which will become the Xa, more frequently appeared as a doublet (12 of 15 SD cells, *p* = 0.02). These observations suggest that, as in ES cells, the two *Xic* loci in cells of early embryos adopt distinct configurations that are regulated by *Xist* and correlate with fate. Thus, the chromosome states reflected by FISH may play a role in the initial random designation of the Xa and Xi in vivo.

After one X chromosome is silenced, it remains the Xi throughout all subsequent cell divisions. We analyzed differentiated cells to determine whether SIAR persists after X-inactivation is established. In MEFs, the X-chromosomal loci *Mecp2* and the *Xic* exhibited significantly reduced proportions of SD signals when compared to ES cells ( Figures 5A and S4A), and the proportions of SD signals were not altered upon MeOH fixation ( Figures 5C and S4B), indicating that MEFs do not exhibit SIAR. A second differentiated cell population, trophectoderm cells from blastocyst-stage embryos, showed a proportion of SD *Xic* signals comparable to that seen in MEFs and significantly lower than that seen in ES cells ( Figure 5A). Taken together, these observations indicate that SIAR occurs only in cells that are poised for X-inactivation. Furthermore, they suggest that SIAR does not reflect a mechanism that maintains the identity of the Xa and Xi in differentiated cells but instead reflects a process that is involved in the initial designation of X chromosome fates.

## Discussion

In this study, we used FISH to demonstrate that the two X chromosomes in female mouse ES cells differ prior to X-inactivation. On one X chromosome, the *Xic* tended to appear as a singlet signal when assayed by FISH and other X-linked genes more often appeared as doublets. The second X chromosome was more likely to show the opposite pattern. In ES cell lines that are destined for nonrandom X-inactivation, the future Xi exhibited a high frequency of singlet signals for the *Xic* and of doublet signals for other X-chromosomal loci, while the future Xa showed the opposite pattern. Taken together, these data suggest that, prior to X-inactivation, the two X chromosomes in a female cell exist in distinct future Xi and future Xa states.

We propose that an absolute difference between the X chromosomes underlies the future Xi and future Xa states. The state of the X chromosome in turn affects the probability that a locus will appear as a singlet or doublet FISH signal. In *Tsix*-pA/+ and Δ *Xist*/+ ES cells, where a given X chromosome will become the Xi in 100% of cells, the *Xic* appeared as a singlet on that chromosome in 70% of SD cells. A simple explanation for this observation is that FISH reflects dynamic behavior of loci, and the future Xi and future Xa differ in their dynamics. For example, the *Xic* may fluctuate between appearing as a singlet or doublet on both the future Xi and the future Xa, appearing more frequently as a singlet on the future Xi and more frequently as a doublet on the future Xa ( Figure S3). In this model, the frequency with which a locus on the future Xi or Xa appears as a singlet or doublet in the population, and not its appearance in a single cell at any given point in time, reveals the underlying state of the chromosome.

In cell lines destined for random and completely nonrandom X-inactivation, the frequency with which a given X chromosome adopts the future Xi state correlates with the frequency with which it will be inactivated. The same relationship was observed in cell lines that will display skewed X-inactivation due to the *Xce* effect. Thus, the *Xce* effect is manifested prior to X-inactivation. This result is consistent with the suggestion that *Xce* effect influences the initial assignment of X chromosome fates [ 26].

Our observation that X chromosomes adopt distinct future Xi and future Xa states suggests that X chromosomes know their fates prior to silencing. These states exist even in cell lines that will undergo random X-inactivation, raising the question of how randomness is achieved. In clonal populations of ES cells that will undergo random X-inactivation, a marked X chromosome showed a singlet signal for an X-linked gene in 50% of cells, suggesting that X chromosomes can switch states.

**Figure 6:**
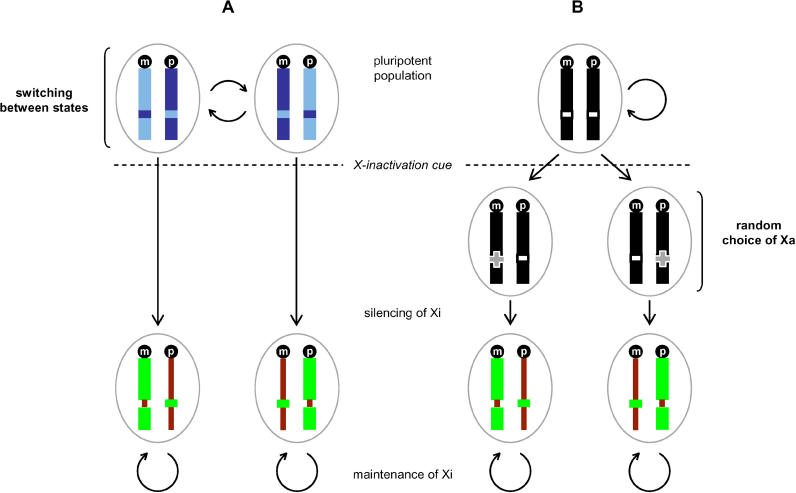
Models for Achieving Randomness in X-Inactivation (A) The data in the present study suggest a model in which the X chromosomes in pluripotent cells (m indicates maternal X chromosome, p indicates paternal X chromosome) coordinately switch between future Xa (light blue with dark blue *Xic*) and future Xi (dark blue with light blue *Xic*) states in cycling cells. The fates of the X chromosomes as the Xa (green with red *Xic*) or the Xi (red with green *Xic*) are determined by their states at the time that the cell receives the cue to initiate X-inactivation. (B) The prevailing model holds that the two X chromosomes in pluripotent cells (black, *Xic* indicated in white) are equivalent until the cue that initiates X-inactivation causes differential marking of the two X chromosomes (gray cross), thus designating the Xa and the Xi.

We propose a model in which switching of X chromosomes between mutually exclusive future Xi and future Xa states is the source of the apparent randomness of X-inactivation ( Figure 6A). In each cell, one X chromosome adopts the future Xi state and the other X chromosome adopts the future Xa state. When a cell receives the cue to initiate X-inactivation, the chromosome that exists in the future Xi state in that cell will be silenced. However, as long as that cell remains pluripotent, the states of the chromosomes are not fixed and can switch in a concerted fashion. Because of this switching, the two X chromosomes each assume the future Xi state in half of the cells in a population. This randomization of states provides the basis for silencing to occur in an apparently random manner when X-inactivation is triggered. In this model, the mechanisms that determine the randomness of X-inactivation function prior to the receipt of the differentiation signal that initiates X-inactivation. In contrast, the prevailing model for randomness of X-inactivation posits that the two X chromosomes are equivalent in pluripotent cells, and that designation of which chromosome will be silenced occurs stochastically upon receipt of the signal that triggers X-inactivation ( Figure 6B) [ 4, 27]. Instead, our data support a model similar to the class of models proposed by Williams and Wu [ 28], who speculate that a switching-based mechanism, like that regulating mating-type switching in fission yeast, may underlie the randomness of X-inactivation.

X-chromosomal loci exhibited SD signals in a significant fraction of MeOH-fixed and of PFA-fixed ES cells. In a recent study, Gribnau et al. [ 11] also observed a high frequency of SD FISH signals for X-chromosomal loci in MeOH-fixed ES cells. Based on the large fraction of cells exhibiting SD signals, it was suggested that X-linked genes are subject to asynchronous replication in ES cells. We directly assayed replication timing of two X-chromosomal loci, *Pgk1* and the *Xic,* in ES cells. Each exhibited a single peak of replication. Cells showing SD FISH signals for the *Xic* or *Pgk1* occurred at a high frequency even after both alleles of these two X-chromosomal loci had replicated. In addition, a FISH signal intensity quantification assay revealed that in individual ES cells, the singlet and doublet signals for the *Xic* or for *Pgk1* contained comparable amounts of DNA. Together, these data indicate that the high frequency of SD FISH signals for X-chromosomal loci in ES cells is not due to highly asynchronous DNA replication ( Figure S5).

In PFA-fixed ES cells, the *Xic* was unusual in that it tended to exhibit a singlet FISH signal while other genes on the same X chromosome more often showed doublet signals. In contrast, the *Xic* and adjacent genes exhibited concordant FISH signals when ES cells were fixed with MeOH ( Figure S6A) [ 11]. Thus, the opposite behavior of the *Xic* was observed only in PFA-fixed ES cells. In addition, when ES cells were fixed with PFA, the future Xi showed a higher frequency of singlet signals for the *Xic* or of doublet signals for other X-linked genes. In contrast, when ES cells were fixed with MeOH, the future Xi and future Xa showed equal probabilities of exhibiting singlet signals for the *Xic* or other X-linked genes ( Figure S6B) [ 11]. PFA fixation maintains nuclear structure while MeOH extracts histones and other chromatin proteins and perturbs nuclear organization [ 19]. Therefore, the differences between PFA- and MeOH-fixed samples suggest that some aspect of chromatin structure or nuclear organization is required for replicated alleles on the future Xi and future Xa to appear as singlets with different probabilities. One possible explanation is that there is differential cohesion between sister chromatids on the two X chromosomes. Whatever the physical basis of the singlet and doublet FISH signals, they demonstrate that the X chromosomes differ prior to X-inactivation in a manner that is predictive of Xi and Xa fates.

The *Xic* behaved oppositely to other loci on both the future Xi and the future Xa, mirroring the opposite expression patterns of *Xist* and most X-linked genes after X-inactivation and suggesting a correlation between the appearance of a locus by FISH and its future expression status. Azuara et al. [ 17] reported a correlation between FISH signal appearance and current expression status. In that study, a replicated transgene tended to exhibit a doublet FISH signal when it was expressed and a singlet FISH signal when it was silenced. In our study, the replicated allele that more often appeared as a singlet signal in ES cells was the one that would be expressed after X-inactivation is triggered by differentiation. This result suggests that organization of sequences within the nucleus of a pluripotent embryonic cell may impact their expression after differentiation.

In wild-type ES cells, X chromosomes appear to switch between states in a concerted manner: when one X chromosome assumes the future Xi state, the other adopts the future Xa state. In heterozygous *Xist* and *Tsix* mutant ES cells, it appears that the X chromosomes no longer switch between states, suggesting that these noncoding RNAs are either required for switching to occur or affect the likelihood that a chromosome will adopt the future Xi or future Xa state each time a switch occurs. The probability that a chromosome will become the Xi (or the Xa) is determined by the opposing activities of *Xist* and *Tsix* on that chromosome [ 29]. These observations suggest that *Xist* and *Tsix* influence the fates of both X chromosomes by determining how effectively each chromosome competes to adopt the future Xi (or future Xa) state prior to X-inactivation. Both *Xist* and *Tsix* RNAs have been implicated in the regulation of chromatin structure [ 30– 32]. Prior to X-inactivation, these noncoding RNAs can be detected exclusively at the *Xic* on both transcriptionally active X chromosomes [ 8]. Perhaps *Xist* and *Tsix* RNA mediate changes in chromatin structure at the *Xic* prior to X-inactivation. Such changes may in turn modulate the state of the entire X chromosome and direct its fate.

Our data show that prior to random X-inactivation, X chromosomal loci differ in a manner that is predictive of future expression status. In addition to X-linked genes, several thousand autosomal genes are also subject to random monoallelic expression. It has been suggested that random monoallelic loci on autosomes and the X chromosome may share regulatory features [ 1, 33]. It will be interesting to determine whether a common mechanism of concerted switching between two states underlies the randomness of monoallelic expression throughout the genome.

## Materials and Methods

### Cell lines and culture.

Wild-type female ES cells [ 34], Δ *Xist*/+ ES cells [ 11, 20], *Tsix*-pA/+ ES cells [ 14], X.2/X ^wt^ ES cells [ 21], and MEFs were cultured according to standard practice. Wild-type and Δ *Xist*/+ [ 20] 3.5-d postconception blastocysts were harvested by standard procedures and cultured overnight in ES medium without LIF. To identify cells in S phase, cells were cultured with BrdU (Amersham Biosciences, Little Chalfont, United Kingdom) for 15 to 30 min.

### Cell cycle fractionation.

Flow cytometry was performed on live ES cells labeled with BrdU (Amersham) and stained with 40 μg/ml Hoechst 33342 (Molecular Probes, Eugene, Oregon, United States) for 45 min prior to harvesting. Cells were resuspended in ES medium plus Hoechst, 7% Cell Dissociation Buffer (GIBCO, San Diego, California, United States), and 10 mM EDTA for sorting and were cooled during the procedure. Cytometry was performed using a FACSDiVa Cell Sorter (Becton-Dickinson, Palo Alto, California, United States). Cell cycle profiles were generated by excitation with a violet laser; Hoechst emission was measured with a HQ445/50 bandpass filter (Chroma Technology, Rockingham, Vermont, United States). Cells were gated to exclude debris and double cells. For microscopy, fractions were sorted into PBS in multiwell slides pretreated with 1 mg/ml poly- l-lysine and allowed to settle and adhere.

### Sample preparation.

ES cells and MEFs were fixed for FISH using PFA [ 12] or MeOH [ 35]. Blastocysts were treated with acid tyrode to remove zona pellucidae, applied to 2% gelatin-coated slides using a Cytospin apparatus (Shandon, Pittsburgh, Pennsylvania, United States), PFA-fixed for 10 min, and permeabilized with a 5-min incubation in PBS plus 0.5% Triton X-100.

### FISH.

BACs used for genomic probes are listed in Table S1. All pairwise DNA FISH was performed on loci separated by 40 Mb or less; linked sequences can be reliably scored as being on the same chromosome over distances of up to 50 Mb [ 33]. A collection of PCR products spanning exon 1 was used to make *Xist* probes. Probes were generated using a BioPrime kit (Invitrogen, Carlsbad, California, United States), or using cy3-dCTP or FITC-dUTP (Amersham; Enzo Life Sciences, Farmingdale, New York, United States) with kit reagents. Strand-specific probes to detect *Xist* and *Tsix* RNA were generated by in vitro transcription with FITC-UTP or bio-CTP (Enzo Life Sciences) from an *Xist* exon 7 template.

FISH for genomic DNA was performed as described [ 35]. Biotinylated probes were detected with FITC-avidin (Vector Laboratories, Burlingame, California, United States) or cy3-streptavidin (Amersham). Combined DNA and RNA FISH was performed as described [ 36], with the addition of a pepsin pretreatment prior to the initial step [ 35]. BrdU detection was performed as described [ 35] using mouse monoclonal α-BrdU antibody (Becton Dickinson) and α-mouse FITC (Vector Laboratories).

Samples were scored on an Olympus BX60 microscope. Images were collected with a Hamamatsu ORCA-ER digital camera using Openlab 4.0.1 software, assembled using Adobe Photoshop 7.0, and levels adjusted to enhance contrast. ICM cells of blastocysts were scored from three-dimensional images collected using a DeltaVision system as described below.

### FISH signal intensity quantification.

Images were collected as 0.1-μm optical section stacks using an Olympus IX70 microscope with a motorized stage controlled by DeltaVision 2.10 software (Applied Precision, LLC, Issaquah, Washington, United States) and a MicroMax CCD camera (Roper Scientific, Tucson, Arizona, United States). SoftWorx 2.50 software was employed to deconvolve three-dimensional images and sum pixel intensity through relevant sections of image stack to generate two-dimensional projections representing total intensity of each FISH signal. The software was allowed to delineate the signal circumference and to integrate pixel intensities to generate an overall intensity value in arbitrary units.

### Mimosine arrest and release replication timing assay.

S-phase time-course experiments were performed using a mimosine arrest-release protocol [ 35]. DNA from each time point was isolated and sonicated as described [ 37]. BrdU-labeled human DNA (0.5 μg) was mixed with 10 μg of DNA from each time point for normalization. Labeled DNA was immunopurified using an α-BrdU monoclonal antibody (Becton Dickinson) and Protein G Sepharose 4 Fast Flow beads (Amersham) and resuspended in 1 ml of 1 mM Tris, 0.1 mM EDTA (pH 8.0). PCR primers are listed in Table S2. For FACS analysis of mimosine arrest/release time points, samples were fixed overnight in 70% ethanol, treated with 0.2 μg/ml RNase, stained with 20 μg/ml propidium iodide (Molecular Probes), and analyzed using an FACSCalibur (Becton Dickinson).

### Statistics.

All *p*-values were determined by comparing the observed distribution of signal patterns at each allele to a random, 50/50 distribution (null hypothesis) using a χ ^2^ distribution test with one degree of freedom.

## Supporting Information

Figure S1 Supporting Data for Figure 1
(A) The proportions of PFA-fixed ES cells exhibiting SS (white), SD (black), and DD (gray) signals at autosomal *(Hba1, Fn1)* and X-chromosomal *(Ccnb3, Hprt, Mecp2, Xic, Pgk1, Grpr)* loci.
Average data from two to four independent experiments ( *n* ≥ 150) are shown. SD fractions from these data are presented in Figure 1B.
(B) FACS analysis of mimosine arrest/release time-course samples from the experiment shown in Figure 1C illustrates release of the majority of cells and synchrony of progression through S phase. Samples collected at 2-h intervals, starting at 1 h after release and continuing until 11 h postrelease, were analyzed by propidium iodide (PI) staining for DNA content.
(119 KB PPT)

Figure S2 Allele-Specific FISH for the *Xic* (red) in 129-tet/ *cas* ES Cells for the Experiment Scored in Figure 4C
Shown are the two classes of SD cells in which the 129-tet allele, identified using a tet-operator probe (green; yellow overlap, indicated by white arrowheads), appears as the singlet (left) or the doublet (right).
(269 KB PPT)

Figure S3 A Dynamic Model for SIAR (A) We hypothesize that after replication, both alleles of an X-chromosomal locus fluctuate between appearing as a singlet and a doublet FISH signal, and that one allele preferentially appears as a doublet (navy), while the homologous allele preferentially appears as a singlet (aqua). Schematics show the four possible FISH signal patterns for a single locus (SS, SD or DS, DD); the approximate percentages of cells displaying each pattern for the *Xic* are indicated. SD/DS FISH signals are subdivided into the approximate percentages in which the singlet is displayed by the aqua allele vs. the navy allele, based on allele-specific FISH in Δ *Xist*/+ and *Tsix*–pA/+ ES cells ( Figure 3C). Several observations are consistent with this model. First, SS, SD, and DD populations coexisted at apparent equilibrium ( Figure 1D, fractions 3–5) until chromosome condensation at G2/M ( Figure 1D, fraction 6), suggesting that loci may interconvert between appearing as singlet and doublet FISH signals. Second, most X-linked loci on one X chromosome exhibited approximately 65% concordance when analyzed pair-wise, showing singlet signals for both loci on one chromosome and doublet signals for both loci on the other chromosome ( Figure 2C). This degree of concordance can be accounted for by the independent fluctuation of linked loci, which all exhibit the same probability of appearing as a singlet along a given chromosome. Third, most loci on the future Xa in Δ *Xist*/+ and *Tsix*–pA/+ ES cells exhibited an elevated frequency of appearing as singlet signals. However, these loci did not appear as singlets in every cell, consistent with loci fluctuating between appearing as a singlet or a doublet and spending more time as a singlet on the future Xa.
(B) Calculation of the probabilities with which alleles on each chromosome appear as singlets or doublets in the dynamic model described above. The frequencies with which the navy and aqua alleles appear as a singlet or doublet signal are described by the terms pS and pD, where pD = 1 − pS. The table lists pS and pD values estimated from our data. We assumed that the identities of the future Xa and future Xi are fixed in Δ *Xist*/+ and *Tsix*–pA/+ ES cells, and that for the *Xic* locus, the navy allele (low pS, mostly doublet) occurred on the future Xa and the aqua allele (high pS, mostly singlet) occurred on the future Xi. The overall frequency of SS (approximately 20%), SD (approximately 50%), DD (approximately 30%) FISH signals in the wild-type population were considered. For the navy allele, the pS was calculated by adding the fraction of SS signals (where both the navy and aqua alleles appeared as singlets) to the fraction of SD signals in which the navy allele appeared as the singlet (as determined from the Δ *Xist*/+ and *Tsix*–pA/+ cell lines, in which it appeared as the singlet in approximately 30% of SD signals). A similar calculation was performed for the aqua allele.
(C) Illustration of the calculation for the frequency with which an allele fixed in the aqua state (high pS) will appear as the singlet in a cell exhibiting an SD signal at a given locus. Calculations were based on the values listed in Figure S3B. The calculation shows that strict differences in the underlying states of the X chromosomes could result in the observed approximately 69% bias in singlet allele identity seen in heterozygous *Xist* and *Tsix* mutant ES cells ( Figure 3C).
(D) Prediction of FISH signal concordance frequencies for two linked loci that inhabit the same state (both navy on one chromosome, both aqua on the other) and that are fluctuating independently. Even though states are strictly coordinated on each homolog under the model, the predicted frequency of concordant SD SD FISH signals is 58%, comparable to the observed frequency of 62% to 65% ( Figure 2C).
(E) Use of the model to predict the degree of skewing in singlet allele identity in 129-tet/ *cas* ES cells. The proportion of the time that the 129 *Xic* allele is expected to appear as the singlet in SD cells is calculated based on the results of Figure S3C. The model assumes that the 129 *Xic* allele is in the aqua state in 80% of cells, and in the navy state in the remaining 20% of cells. The model predicts 62% skewing toward the 129 allele appearing as the singlet signal, which is in close agreement to the 60% skewing observed ( Figure 5C).
(71 KB PPT)

Figure S4 MEFs Do Not Display SIAR (A) Comparison of the proportion of PFA-fixed S-phase (BrdU+) ES cells (dark gray) and MEFs (light gray) exhibiting SD signals for an autosomal locus *(Hba1)* and two X-chromosomal loci *(Xic*, *Mecp2)*.
Data represent two or three independent experiments.
(B) Distribution of FISH signal classes (SS, white bars; SD, black bars; DD, gray bars) in S-phase (BrdU+) MEFs fixed with PFA (upper) or MeOH (lower) and probed for *Hba1,* the *Xic,* and *Mecp2*. Representative experiments are plotted.
(77 KB PPT)

Figure S5 Summary of Replication Timing Data for the *Xic*
(A) Table summarizing data pertaining to the replication timing of the *Xic* in ES cells and fibroblasts
Data from the present study are represented in columns 1 ( Figure 5A), 2 (upper: Figure 5C; lower: Figure 1F), 3 (lower: Figure 1C), 5 ( Figure S5B), and 6 ( Figure 1E). Determination of *Xic* replication timing in fibroblasts using S-phase fractionation followed by immunopurification of BrdU-labeled material and PCR analysis (column 3, upper) was reported by Hansen et al. [ 38]. Column 4 indicates that fibroblasts, but not ES cells, contain a uniformly late-replicating X chromosome when assayed by immunostaining of chromosome spreads from BrdU-pulsed cells [ 20, 39]. Taken together, these data indicate that the elevated frequency of SD FISH signals observed in MeOH-fixed ES cell samples does not correlate with highly asynchronous DNA replication of the *Xic*.
(B) *Xic* FISH signal intensity quantification as in Figure 1E for MeOH-fixed MEFs and ES cells. S/D FISH signal intensity ratios in individual nuclei displaying SD signals are plotted. The signal intensity ratio distributions are consistent with the interpretations that (1) in MEFs exhibiting an SD pattern, most singlet and doublet signals represent unreplicated and replicated alleles, respectively, and that (2) in ES cells exhibiting an SD pattern, the majority of the singlet and doublet FISH signals reflect equal amounts of DNA at the two alleles.
(50 KB PPT)

Figure S6 Coordination and Absence of Correlation with Fate in MeOH-Fixed Cells (A) Coordination of FISH signals for X-chromosomal loci in MeOH-fixed ES cells. Frequencies of concordance and discordance for specified locus pairs were scored as in Figure 2B. *p*-Values reflect the probability that the observed distributions are random. A high *p*-value for *Mecp2* and the *Xic* reflects the small sample size. As previously reported, the *Xic* exhibited a high proportion of concordant FISH signals with the linked *Mecp2* gene in samples fixed with MeOH [ 11], whereas in PFA-fixed samples this locus pair exhibited predominantly discordant FISH signals ( Figure 2C).
(B) Scoring of allele-specific FISH in MeOH-fixed *Tsix*–pA/+ ES cells. Cells displaying an SD pattern for the *Xic* were scored for identity of the allele displaying the singlet signal as in Figure 3B. *p*-Values reflect the probability that the observed distributions are random. This analysis supports the conclusion that in MeOH-fixed ES cells, the *Xic* on the future Xi does not display an increased likelihood of exhibiting a singlet signal [ 11], in contrast to what was seen in PFA-fixed ES cells ( Figure 3C).
(33 KB PPT)

Table S1 BACs Used as FISH Probes BACs were obtained from the BACPAC Resources Center at Children's Hospital Oakland Research Institute ( http://bacpac.chori.org). BACs included the genes indicated on the left side of the table.
(24 KB PPT)

Table S2 PCR Primers Used to Assay Immunopurified BrdU-Containing DNA from Mimosine Arrest-Release Time-Course Fractions ( Figure 1C)
Standard PCR conditions were used for 34 to 39 cycles, with the exception that annealing time was extended for the first seven cycles.
(30 KB PPT)

## Abbreviations

BrdU: 5-bromo-2-deoxyuridine
D: doublet FISH signal
ES: embryonic stem
FISH: fluorescence in situ hybridization
ICM: inner cell mass
MEF: mouse embryo fibroblast
MeOH: methanol:acetic acid
PFA: paraformaldehyde
S: singlet FISH signal
SIAR: SD FISH signals that are independent of asynchronous DNA replication
Xa: active X chromosome
Xi: inactive X chromosome
*Xic*: *X-inactivation center*
